# Antimicrobial Activity of Cultivable Endophytic and Rhizosphere Fungi Associated with “Mile-a-Minute,” *Mikania cordata* (Asteraceae)

**DOI:** 10.1155/2020/5292571

**Published:** 2020-06-15

**Authors:** Pavithra L. Jayatilake, Helani Munasinghe

**Affiliations:** Department of Botany, University of Sri Jayewardenepura, Nugegoda 10250, Sri Lanka

## Abstract

Endophytic and rhizosphere fungi are understood to be aiding the host plant to overcome a range of biotic and abiotic stresses (nutrition depletion, droughts, etc.) hence, they remain to be reservoirs of plethora of natural products with immense use. Consequently, this investigation of endophytic and rhizosphere fungi isolated from *Mikania cordata* (a perennial vine that is well established in Sri Lanka) for their antimicrobial properties was performed with the aim of future derivation of potential beneficial pharmaceutical products. Leaves, twigs, and roots of *M. cordata* were utilized to isolate a total of 9 endophytic fungi out of which the highest amount (44%) accounted was from the twigs. A sample of the immediate layer of soil adhering to the root of *M. cordata* was utilized to isolate 15 rhizosphere fungi. *Fusarium equiseti* and *Phoma medicaginis* were endophytes that were identified based on colony and molecular characteristics. The broad spectrum of antimicrobial activity depicted by *F. equiseti* (MK517551) was found to be significantly greater (*p* ≤ 0.05, inhibitory against *Bacillus cereus* ATCC 11778, *Staphylococcus aureus* ATCC 25923, *Escherichia coli* ATCC 25922, and *Pseudomonas aeruginosa* ATCC 25853) than *P. medicaginis* (MK517550) (inhibitory against *Staphylococcus aureus* ATCC 25923, *Escherichia coli* ATCC 25922, and *Pseudomonas aeruginosa* ATCC 25853) as assessed using the Kirby-Bauer disk diffusion method. *Trichoderma virens* and *Trichoderma asperellum* were rhizospere fungi that exhibited remarkable antimicrobial properties against the test pathogens chosen for the study. *T. asperellum* indicated significantly greater bioactivity against all four bacterial pathogens and *Candida albicans* ATCC 10231 under study. The ranges of minimum inhibitory concentrations (MICs) of the fungi depicting antimicrobial properties were determined. The results obtained suggest that *F. equiseti*, *P. medicaginis*, *T. asperellum*, and *T. virens* of *M. cordata* harness bioprospective values as natural drug candidates. This is the first report on isolation and evaluation of the antimicrobial properties of endophytic and rhizosphere fungi of *Mikania cordata.*

## 1. Introduction

The exacerbation of drug resistance towards numerous commercially available antibiotics by many existing pathogenic fungi and bacteria has piqued the discovery of novel therapeutics rather important and timely. The major reasons for the successful evolution of antibiotic resistance are the overuse and abuse of antibiotics [1–3]. Inappropriate prescription, lack of new antibiotics, and regulatory barriers for research also play a hand in increasing resistance. The process of horizontal gene transfer ensures the accumulation of genes of resistance in between species.

Endophytic and rhizosphere fungal microbiota are found to contain a high metabolic capability in terms of producing a myriad of secondary metabolic products (peptaibols, diketopiperazines, sesquiterpenes, steroids, etc.) which can be exploited as antimicrobials, anti-inflammatory agents, antitumour agents, antioxidants, and even plant growth-promoting agents [4]. The quantities and types of secondary metabolites produced by the fungi residing in plant tissues and those found in the plant's rhizospheric soils could depend on a cluster of biotic and abiotic factors such as the microbiome-host interactions, humidity, environmental temperature, type of soil, and quality of root exudates [5]. It has been estimated that only about 1-2% of the entire set of 300,000 identified plants have still been studied for their endophytic microbial communities. The composition of endophytes and their relationship to the species of the host have not yet been completely understood. It is hypothesized that it could occur by chance and then reside for a long time based on the conditions that are prevailing within the host tissues and the external environment [6, 7].

The present study was conducted with the aim of discovering potential novel bioactive antimicrobial compounds from a wide range of natural resources, which are the associated endophytic and rhizosphere fungi of the terrestrial plant, *Mikania cordata* (Asteraceae). *Mikania cordata* is native to Central and South America and is an immensely used candidate in the field of traditional medicine over generations. It is known that the raw extract of leaves of *M. cordata* plant is widely used to treat eye sores, scorpion and snake bites, coughs, and various gastrointestinal infections [8, 9]. The leaf pulp is commonly used as poultice for open wounds which will cause efficient healing. The decoction of *M. cordata* leaves is used in treating ulcers and dysentery [10]. An ointment formulated from the leaf extract of *M. cordata* possessed *in vitro* antibacterial activity against methicillin-resistant *Staphylococcus aureus* and antifungal properties against *Trichophyton mentagrophytes* [11]. However, *M. cordata* is also considered as a weed, owing to its extremely fast-growing nature. It is also known as “mile-a-minute.” The traditional practice is to destroy the vine mesh at the onset of the flowering season because if left undisturbed, it could be a devastating weed [12]. The antimicrobial properties of the *M. cordata* leaf extracts have been reported before, but its endophytic and rhizosphere fungal communities have not yet been examined for their bioactive potentials.

## 2. Materials and Methods

### 2.1. Collection of Plant Material

Healthy, fresh, and randomly selected twenty-five *Mikania cordata* plant specimens containing roots, twigs, leaves, and stems were collected from Sri Jayewardenepura Kotte, Sri Lanka (6°54′8.218^″^N, 79°54′15.152^″^E). Samples were brought to the laboratory in clean plastic bags and were utilized in experimental purposes within 24 h. The voucher herbarium specimen was prepared [13], and the authentication was carried out by the National Herbarium of the Royal Botanical Gardens, Peradeniya, Sri Lanka.

### 2.2. Isolation of Endophytic Fungi

A standard protocol was followed with few modifications [14]. The plant parts containing roots, twigs, stems, and leaves were carefully washed under running tap water to remove adhered soil particles, dust, and epiphytes. Regular-sized pieces were carefully cut from the leaves (1 cm × 1 cm), twigs (1 cm), and root (1 cm) samples using a sharp sterilized scalpel. The surface disinfection procedure included the pieces of samples being dipped in 5% (*v*/*v*) sodium hypochlorite for 3 minutes. They were then washed with sterile distilled water (SDW) for 1 min, and it was repeated twice. The samples were then dipped in 70% (*v*/*v*) ethanol for 1 min and were washed with SDW for 1 min. The final step was repeated thrice.

The surface-disinfected leaf samples were placed on Potato Dextrose Agar (PDA) enriched with Chloramphenicol (50 mg/l). The twig and root samples were split longitudinally and were placed on PDA enriched with Chloramphenicol (50 mg/l). The incubation was done at 28 ± 2°C for 4-6 days. Pure cultures of the endophytic fungi were obtained by transferring the hyphal tips onto fresh PDA plates.

The effectiveness of the surface disinfection was tested by the tissue imprinting procedure. Surface-disinfected leaf samples were transferred to fresh PDA enriched with antibiotics. It was left for 30 min to obtain the imprints, and the segments were removed and incubated at 28 ± 2°C for three to five days. A similar procedure was followed for twig and root samples.

The controls were portions of leaf, twig, and root samples that were not subjected to the surface disinfection protocol.

### 2.3. Isolation of Fungi from *M. cordata* Rhizosphere

The *M. cordata* vine was uprooted along with intact soil (6°54′8.218^″^N, 79°54′15.152^″^E), and they were transported to the laboratory in sterile polythene bags. The soil that adhered to the roots was carefully scraped using a sterilized spatula and weighed aseptically [15]. The soil dilution plate method was carried out to isolate the rhizosphere fungi. The weighed soil (1 g) was transferred to an Erlenmeyer flask containing 10 ml of SDW. The flask was kept on the shaker at 150 rpm for 5 min. The supernatant was used to prepare the 10-fold dilution series up to 10^−5^. The 10^−3^, 10^−4^, and 10^−5^ dilutions were used for the isolation of rhizosphere fungi so as to avoid overcrowding. The Petri dishes containing PDA enriched with Chloramphenicol (50 mg/l) were spread with 100 *μ*l of each dilution in triplicate. The plates were incubated at 28 ± 2°C for 4-6 days. The emerging fungal colonies were subcultured into fresh PDA and incubated at 28 ± 2°C for 5 days. Pure cultures were obtained by transferring the hyphal tips onto fresh PDA.

### 2.4. Test Pathogenic Microorganisms

Standard cultures of a representative set of human pathogenic microorganisms, including gram-positive bacteria (*Bacillus cereus* ATCC 11778, *Staphylococcus aureus* ATCC 25923), gram-negative bacteria (*Escherichia coli* ATCC 25922, *Pseudomonas aeruginosa* ATCC 25853), and nonfilamentous fungi (*Candida albicans* ATCC 10231, *Candida tropicalis* ATCC 13803, and *Candida parapsilosis* ATCC 22019), were utilized.

### 2.5. *In Vitro* Preliminary Screening of Endophytic and Rhizosphere Fungi for Antimicrobial Activity

All the isolated endophytic and rhizosphere fungi were subjected to an agar plug diffusion assay [16]. The lawns of test bacterial pathogens were prepared on Mueller Hinton Agar (MHA), and those of yeast pathogens were prepared on Sabouraud Dextrose Agar (SDA) using sterile cotton swabs. A sterile cork borer was used to obtain agar plugs (6 mm diameter) of actively growing pure cultures of fungi in PDA not enriched with Chloramphenicol. They were transferred to the media seeded with test pathogenic microorganisms (turbidity of 0.5 McFarland standards) in triplicate and were incubated at 37°C for 24 h. The mean diameter of the zones of inhibition (ZOI) was obtained post incubation.

### 2.6. Identification of the Bioactive Endophytic and Rhizosphere Fungi

The two endophytic fungi (MCEF001 and MCEF002) and two rhizosphere fungi (MCRF003 and MCRF006) were chosen for the secondary screening and determination of the minimum inhibitory concentration (MIC) based on the wide spectrum of results obtained from the preliminary screening.

Fungal identifications were based on the morphological and molecular characteristics.

Extraction of genomic DNA (gDNA) was performed [17] and was subjected to PCR amplification. ITS-1 forward primer (5′TCC GTA GGT GAA CCT GCG G 3′) and ITS-4 reverse primer (5′ TCC TCC GCT TAT TGA TAT GC 3′) were utilized for the purpose of amplifying the ITS region of the fungi according to a published protocol [18] with few modifications of the volumes of the reagents used.

The master mixture needed for the PCR reactions was prepared so that the total volume per sample was 15 *μ*l. It was a consortium of 1.5 *μ*l of 10x DreamTaq™ Green buffer (with 20 mM MgCl_2_, loading dye, Tris HCL maintaining pH at 8.5), 0.6 *μ*l dNTPs (Genetech equimolar of 10 mM dATP, dGTP, dTTP, and dCTP), 0.6 *μ*l of ITS-1 forward primer (10 mM stock solution), 0.6 *μ*l of ITS-4 reverse primer (10 mM stock solution), Taq DNA polymerase (5 U/*μ*l) (Sigma), 9.72 *μ*l of sterile deionized water, and 1.8 *μ*l of the DNA template. Hence, 13.2 *μ*l of the master mix was added with 1.8 *μ*l of the DNA template. The DNA template was prepared by diluting the gDNA working solution by 10-folds using nuclease-free water. The positive control was the 10-fold diluted gDNA of *Fusarium oxysporum*, and the negative control was 1.8 *μ*l of sterile deionized water instead of the DNA template.

PCR amplification was carried out in a thermocycler (Bio-Rad® T100TM thermocycler, USA). The initial denaturation step at 94°C was carried out for 5 min which was followed by 35 cycles of denaturation at 94°C for 30 sec, an annealing step at 49°C for 30 sec, and an extension step at 72°C for 1 min. The final extension step at 72°C for 5 min was carried out at the end of the previous 35 cycles.

The specificity of the PCR products was examined by carrying out gel electrophoresis via 2% agarose gel.

The properly amplified PCR products were sequenced by Macrogen, Inc. The BLASTn tool was utilized to acquire the identity of the endophytic fungi by aligning the contig sequences to those found on the NCBI database. NCBI GenBank accession numbers for the DNA sequences of the endophytic and rhizosphere fungi were obtained.

### 2.7. Submerged Fermentation of Endophytic and Rhizosphere Fungi and the Extraction of Secondary Metabolites

The crude extracts of fermented culture broths were prepared [19, 20]. Pure culture of MCEF001 growing in PDA unenriched with Chloramphenicol was used to obtain three mycelial culture plugs (0.5 × 0.5 cm^2^) that were inoculated into 150 ml of sterile Potato Dextrose Broth (PDB) and were incubated at 28 ± 2°C for two weeks on a shaker at 150 rpm.

A double-layered muslin cloth was used to filter the culture broth so as to separate the filtrate from the mycelial mat. The culture filtrate was centrifuged at 4000 rpm at room temperature for 15 min. The mycelia free culture filtrate was then added with 150 ml of ethyl acetate (EA) in a separation funnel and was shaken gently. It was then left stationary for 1 h, and the EA layer was collected. The procedure was repeated twice more, and all three fractions were pooled and concentrated using an angular rotary evaporator (Buchi R-124, Switzerland) (150 rpm at 38°C).

A similar procedure was followed to prepare the EA fractions of the culture broths from MCEF002, MCRF003, and MCRF006.

### 2.8. *In Vitro* Screening of Antimicrobial Activity of Fungal Crude Extracts

The crude extracts of MCEF001, MCEF002, MCRF003, and MCRF006 were tested in triplicate against the test pathogenic microorganisms using the Kirby-Bauer disk diffusion method [21] with each disk containing 30 *μ*l/disk (positive control—Chloramphenicol 30 *μ*g/disk for *S. aureus* and *B. cereus*, Gentamycin 10 *μ*g/disk for *P. aeruginosa* and *E. coli*, Fluconazole 25 *μ*g/disk for *C. albicans*, and Ketoconazole 15 *μ*g/disk for *C. parapsilosis* and *C. tropicalis*. Negative control—EA). Crude extracts were transferred to disks using EA solution.

The mean diameter of ZOI was obtained in triplicates post incubation.

### 2.9. Determination of Minimum Inhibitory Concentrations (MICs)

Crude EA fraction of MCEF001-fermented culture broth was chosen to determine the MIC against *S. aureus*, *E. coli*, *P. aeruginosa*, and *C. parapsilosis* while that of MCEF002 was chosen to determine the MIC against *B. cereus*, *S. aureus*, *E. coli*, *P. aeruginosa*, and *C. parapsilosis*. The MIC ranges of MCRF003 and MCRF006 against *B. cereus*, *S. aureus*, *E. coli*, *P. aeruginosa*, and *C. albicans* were evaluated based on the results obtained for the *in vitro* secondary screening of antimicrobial properties.

The 7.8 mg of EA fraction of MCEF001 was dissolved in 1175 *μ*l of double-strength Mueller Hinton Broth (×2 MHB) to prepare a working solution of 6.5 mg/ml. The dissolution was aided by adding 25 *μ*l of 1% analytical grade dimethyl sulfoxide (DMSO) (*v*/*v*). Similarly, a 6.0 mg/ml working solution was prepared by the EA fraction of MCEF002.

The 7.0 mg of EA fraction of MCRF003 was dissolved in 980 *μ*l of ×2 MHB to prepare a working solution of 7.0 mg/ml. The dissolution was aided by adding 20 *μ*l of 1% analytical grade DMSO (*v*/*v*). Similarly, a 7.2 mg/ml working solution was prepared by the EA fraction of MCRF006.

The broth microdilution method performed using sterile 96-well microdilution plates was used to determine the MIC ranges [22, 23].

Positive control:

- Chloramphenicol for *S. aureus* and *B. cereus*
- Gentamycin for *P. aeruginosa* and *E. coli*
- Ketoconazole for *C. parapsilosis*
- Fluconazole for *C. albicans*

The ranges of MICs of relevant pathogens were recorded according to inhibition of the visible growth by the crude extracts of the endophytic fungi. The assays were triplicated.

### 2.10. Statistical Analysis

Minitab 17 was used to perform one-way ANOVA and pairwise Tukey tests. The results/data were considered significantly different given that *p* < 0.05.

## 3. Results and Discussion

A total of 9 fungal endophytes were isolated from leaves, twigs, and roots of *M. cordata* which depicted morphologically different colony characteristics. The absence of epiphytes or surface-adhering microorganisms was confirmed from the tissue imprint procedure which indicates that the surface disinfection was complete. Effectiveness of surface disinfection could be reassured by culturing aliquots of water from the final washing step on PDA enriched with antibiotics [24]. Surface disinfection is a vital step in isolation of endophytes. The type of disinfectant used, concentration, and its immersion time vary among different tissue samples. Therefore, an optimized protocol could be developed based on thorough literature survey and trial and error [25].

The endophytic fungi isolated from the twigs accounted for more than 44% of the total number of isolates while those isolated from the roots accounted for 22% of the total number of endophytes that were isolated. Three endophytic fungi were isolated from the leaves. However, two of the isolated endophytic fungi (MCEF001 and MCEF002) distinctly depicted broad spectrum antimicrobial activities against gram-positive and gram-negative bacteria and *C. parapsilosis* (Table 1). They were carefully analyzed further. Endophytic fungi are known to produce bioactive molecules belonging to several biochemical classes some of which are phenols, alkaloids, quinones, and flavanoids. These variations of the chemical structures were examined to be the root cause of antimicrobial susceptibilities by different pathogens up to varying extents [26]. The standard of ranges of diameters of the inhibition zones for the disk diffusion method was followed according to CLSI standards [27, 28].

Based on morphological and molecular identifications and BLAST similarities, the endophytic fungi MCEF001 and MCEF002 were identified as *Phoma medicaginis* (GenBank accession number MK517550) and *Fusarium equiseti* (GenBank accession number MK517551 with 99% similarity to the type sequence NR_121457.1). The two isolates depicted 97% and 98% query coverage with 0.0 *E* value, respectively.

All four bacterial pathogens under study were susceptible to the EA fraction of the fermented culture broth of *F. equiseti* while *C. parapsilosis* depicted resistance (Table 2). Our results are in conformance to those observed in a study [29] where a polyketide fusaequisin A extracted from *F. equiseti* was isolated as an endophyte of *Ageratum conyzoides.* It has exhibited inhibitory effects on *S. aureus* and *P. aeruginosa. Fusarium* spp. also are capable of producing commercially important drug precursor PTOX and beauvericin and subglutinol A and B which are antimicrobial compounds [30]. The bacterial pathogens, except for *B. cereus*, were susceptible to the crude EA fraction of the fermented culture broth of *P. medicaginis* while *C. parapsilosis* depicted resistance. The degree of antimicrobial activity exhibited by the EA fraction of *F. equiseti* was significantly greater than that of *P. medicaginis* against *E. coli* and *P. aeruginosa* while that of the EA fractions of both *P. medicaginis* and *F. equiseti* showed no significant difference in action against *C. parapsilosis* (Table 2).

The ranges of MICs (Table 3) observed for EA fraction of *F. equiseti*, against the four bacterial pathogens, are lower than that observed for EA fraction of *P. medicaginis*. Along with the results obtained in Table 2, this suggests that *F. equiseti* endophytic fungus has a comparatively stronger antimicrobial activity against the four test bacteria considered than *P. medicaginis*.

In the current study, a total of 15 rhizosphere fungi were isolated, and 6 out of them depicted antimicrobial activity against the test pathogens. Broad spectrum activity was observed in many of the isolates, but the antimicrobial activity of two rhizosphere fungi stood out among the rest owing to their ability to inhibit at least six of the test pathogenic microorganisms (Table 4). The isolate MCRF006 showed distinct antimicrobial activity against all seven test organisms.

The fungi MCRF003 and MCRF006 were identified as *Trichoderma* spp. based on the morphological data, owing to their characteristic phialides. The isolate MCRF003 was identified as *Trichoderma virens* with an identity of 99%, query coverage of 100%, and an *E* value of 0.0 (GenBank accession number MK517548) while MCRF006 was identified as *Trichoderma asperellum* with a 99% identity, 100% query coverage, and an *E* value of 0.0 to the Type material NR 130668.1 (GenBank accession number MK517549).

We discovered that all four bacterial test organisms were susceptible to the crude EA fraction of *Trichoderma asperellum* while only *S. aureus*, *E coli*, and *P. aeruginosa* were susceptible to the EA fraction of *Trichoderma virens.* Furthermore, the EA fraction of *Trichoderma virens* intermediately inhibited *C. albicans.* Both *C*. *parapsilosis* and *C*. *tropicalis* indicated resistance towards the two rhizosphere fungi under study. The antimicrobial effect of *Trichoderma asperellum* on the four bacterial pathogens was significantly greater than that of *Trichoderma virens* (Table 5).

*Trichoderma* spp. possess the capability of producing over 100 types of secondary metabolites which are antimicrobial in nature. These include compounds of amino acid derivatives, terpenes, pyrones, and polyketides out of which the first identified antibiotic of *Trichoderma* spp. was paracelsin (*α*-aminoisobutyric acid containing peptide isolated from *Trichoderma reesei*) [31].

The range of MIC (Table 6) was determined against the test microorganisms that were susceptible or showed intermediate inhibition for *Trichoderma virens* and *Trichoderma asperellum*. The results indicate that the MIC ranges observed for *Trichoderma asperellum* were comparatively lower than those of *Trichoderma virens*. This may perhaps be due to the ability of *Trichoderma asperellum* isolate to produce higher concentrations of similar bioactive compounds to those of *Trichoderma virens* or due to the presence of a strong group of different antimicrobial compounds.

It has been reported that the antimicrobial activity of *Trichoderma virens* when present as an endophyte and the MIC value obtained against *S. aureus and E. coli* were within the range of 0.128-0.256 mg/ml [32]. However, the contrasting results obtained in the present study may be due to the different host plants and varied conditions in the rhizosphere that changes the composition and concentration of the antimicrobials produced by *Trichoderma virens*.

## 4. Conclusions

This is the first study to explore endophytic and rhizosphere fungi of *Mikania cordata* and evaluate their potential *in vitro* antimicrobial activities. Our results demonstrate that the endophytic *Fusarium equiseti* is capable of depicting a higher antimicrobial activity when compared with *Phoma medicaginis*. *F. equiseti* was found to be effective against all four bacterial pathogens under study while *P. medicaginis* was effective against three bacterial pathogens. The ethyl acetate crude fraction of the culture broth of the rhizosphere fungus *Trichoderma asperellum* was comparatively more effective than that of *Trichoderma virens.* Therefore, the described endophytic and rhizosphere isolates constitute the potential of being attractive sources of pharmaceuticals. The confirmations could be made after cytotoxicity levels and chemical characterizations are verified.

## Figures and Tables

**Table 1:** Preliminary screening of *in vitro* antimicrobial activity of isolated endophytic fungi against the selected test microorganisms by an agar plug diffusion assay performed on MHA (for bacterial pathogens) and SDA (for pathogenic yeasts) media, incubated at 37°C for 24 h. + = presence of a zone of inhibition; − = absence of a zone of inhibition.

Isolated endophytic fungi	Test organism						
	*B. cereus*	*S. aureus*	*E. coli*	*P. aeruginosa*	*C. albicans*	*C. parapsilosis*	*C. tropicalis*
MCEF001	**−**	**+**	**+**	**+**	**−**	**+**	**−**
MCEF002	+	+	+	+	**−**	+	**−**
MCEF003	+	**−**	**−**	**−**	**−**	**−**	**−**
MCEF004	**−**	**−**	+	+	**−**	+	**−**
MCEF005	**−**	+	**−**	+	**−**	**−**	**−**
MCEF006	**−**	+	**−**	**−**	**−**	**−**	+

**Table 2:** Screening of *in vitro* antimicrobial activity of metabolites extracted into the crude EA fraction from PDB of endophytic fungi performed on MHA (for bacterial pathogens) and SDA (for yeast pathogens).

Test pathogenic organism	Mean diameter of the ZOI ± SD (mm) for crude EA extracts (*n* = 3)		Mean diameter of the ZOI ± SD (mm) for positive control (*n* = 3)		
	*P. medicaginis*	*F. equiseti*	Chloramphenicol (30 *μ*g/disc)	Gentamycin (10 *μ*g/disc)	Ketoconazole (15 *μ*g/disc)
*B. cereus*	—	20.3 ± 0.6^B^	22 ± 0^A^	—	—
*S. aureus*	16 ± 0^B^	18 ± 1^B^	28 ± 1^A^	—	—
*E. coli*	19.3 ± 0.6^C^	20.7 ± 0.6^B^	—	22 ± 0^A^	—
*P. aeruginosa*	17.1 ± 0.2^C^	23 ± 0^B^		30 ± 0^A^	
*C. parapsilosis*	16 ± 1^B^	16.7 ± 0.6^B^	—	—	30 ± 0^A^

Mean values sharing common letters in each row are not significantly different *p* ≤ 0.05.

**Table 3:** Range of MIC determined for the crude EA extracts of the culture broths of endophytic fungi (*P. medicaginis* and *F. equiset*i) against human pathogenic microorganisms by the broth microdilution method performed at 37°C for 24 h.

Test pathogenic organism	Range of MIC (mg/ml)	
	*P. medicaginis*	*F. equiseti*
*B. cereus*	—	0.35 > MIC > 0.15
*S. aureus*	1.0 > MIC > 0.45	0.35 > MIC > 0.15
*E. coli*	1.0 > MIC > 0.45	0.35 > MIC > 0.15
*P. aeruginosa*	1.0 > MIC > 0.45	0.35 > MIC > 0.15
*C. parapsilosis*	1.0 > MIC > 0.45	1.5 > MIC > 0.35

**Table 4:** Preliminary screening of *in vitro* antimicrobial activity of isolated rhizosphere fungi against the selected test microorganisms by the agar plug diffusion assay performed on MHA (for bacterial pathogens) and SDA (for pathogenic yeasts) media, incubated at 37°C for 24 h. + = presence of a zone of inhibition; − = absence of a zone of inhibition.

Code numbers of the isolated rhizosphere fungi	Test organism						
	*B. cereus*	*S. aureus*	*E. coli*	*P. aeruginosa*	*C. albicans*	*C. parapsilosis*	*C. tropicalis*
MCRF001	**+**	−	−	−	−	−	−
MCRF003	+	+	+	+	+	−	+
MCRF005	−	−	+	−	−	+	−
MCRF006	+	+	+	+	+	+	+
MCRF007	−	+	−	−	−	−	+
MCRF009	+	+	−	−	−	−	−

**Table 5:** *In vitro* screening of antimicrobial activity of metabolites extracted into the crude EA fraction from PDB of rhizosphere fungi performed on MHA (for bacterial pathogens) and SDA (for yeast pathogens).

Test pathogenic organism	Mean diameter of the ZOI ± SD (mm) for crude EA extracts (*n* = 3)		Mean diameter of the ZOI ± SD (mm) for positive control (*n* = 3)			
	*Trichoderma virens*	*Trichoderma asperellum*	Chloramphenicol (30 *μ*g/disk)	Gentamycin (10 *μ*g/disk)	Fluconazole (25 *μ*g/disk)	Ketoconazole (15 *μ*g/disk)
*B. cereus*	12.3 ± 0.6^C^	17.6 ± 0.6^B^	23 ± 1^A^	—	—	—
*S. aureus*	22.8 ± 0.3^B^	28 ± 1.7^A^	30 ± 0^A^	—	—	—
*E. coli*	15.7 ± 1.2^C^	18.7 ± 0.6^B^	—	22 ± 0^A^	—	—
*P. aeruginosa*	15.3 ± 1.1^C^	22.3 ± 0.6^B^	—	30 ± 0^A^	—	—
*C. albicans*	18.3 ± 0.6^B^	13 ± 1^C^	—	—	44 ± 0^A^	—
*C. parapsilosis*	15.3 ± 0.6^B^	14 ± 1^B^	—	—	—	30 ± 0^A^
*C. tropicalis*	—	14 ± 0^A^	—	—	—	27 ± 0^B^

Mean values sharing common letters in each row are not significantly different *p* ≤ 0.05.

**Table 6:** Range of MIC determined for the crude EA extracts of the culture broths of rhizosphere fungi against human pathogenic microorganisms by the broth microdilution method, incubated at 37°C for 24 h.

Test pathogenic organism	Range of MIC (mg/ml)	
	*Trichoderma virens*	*Trichoderma asperellum*
*B. cereus*	1.15 > MIC > 0.85	1.8 > MIC > 0.9
*S. aureus*	0.85 > MIC > 0.45	0.45 > MIC > 0.2
*E. coli*	1.15 > MIC > 0.85	0.45 > MIC > 0.2
*P. aeruginosa*	1.15 > MIC > 0.85	0.45 > MIC > 0.2
*C. albicans*	0.85 > MIC > 0.45	0.9 > MIC > 0.45

## Data Availability

All data that support the conclusions of this study are described in the article.
